# Preface

**Published:** 1820-06-01

**Authors:** 


### Preface.

### Vol. I. No. 1. JUNE, 1820.

### (ANALYTICAL SERIES.)

Although the arrangement, design, and execution of this Journal are pretty generally known to the Profession, yet the commencement of a New Series, which is destined to travel beyond the range of its predecessors, demands a few prefatory observations, though partly reiterative of formerly declared sentiments.

1mo. The Journal is designed to exhibit a careful, condensed, yet copious Analytical Review of all the principal British Medical Works, with a moderate proportion of select Foreign Publications, intermixed with *practical* commentaries and researches.

2do. No Article in this Review shall proceed from the pen of a young man not practically acquainted with his subject; nor from that of a mere *literary practitioner*, more versed in the indexes of books, than acquainted with the actual phenomena of disease at the bed-side of sickness, and who is consequently incapable of appreciating valuable matter, of rejecting erroneous and useless matter, or of giving wise and safe counsel, in matters of doubt and uncertainty.

3tio. It follows, of course, that this Journal, in aiming to become a granary of clinical facts, practical observations, and legitimate deductions, rejects the mystic reveries of German incubation, the visionary theories of speculative spirits, and the pedantic display of names without things, references without quotations, or quotations without use.

4to. And lastly, since this Journal is free and independent as the air we breathe, so we trust that it will never be *deterred* from doing that which is right, or *seduced* to do that which is wrong.

" Ab honesto, nulla re *deterribitur*—ad turpia nulla spe *invitabitur*."

Seneca.

